# Right drug, right patient, right time: aspiration or future promise for biologics in rheumatoid arthritis?

**DOI:** 10.1186/s13075-017-1445-3

**Published:** 2017-10-24

**Authors:** Vasco C. Romão, Edward M. Vital, João Eurico Fonseca, Maya H. Buch

**Affiliations:** 1 Rheumatology Research Unit, Instituto de Medicina Molecular, Faculdade de Medicina, Universidade de Lisboa, Av. Professor Egas Moniz, 1649-028 Lisboa, Portugal; 2 Department of Rheumatology, Hospital de Santa Maria, Centro Hospitalar Lisboa Norte, Av. Professor Egas Moniz, 1649-035 Lisboa, Portugal; 3 Leeds Institute of Rheumatic and Musculoskeletal Medicine, University of Leeds, Leeds, UK; 4 NIHR Leeds Musculoskeletal Biomedical Research Unit, Leeds Teaching Hospitals NHS Trust, Leeds, UK

**Keywords:** Personalised therapy, Biological therapy, Rheumatoid arthritis, Response predictors, Biomarkers

## Abstract

Individualising biologic disease-modifying anti-rheumatic drugs (bDMARDs) to maximise outcomes and deliver safe and cost-effective care is a key goal in the management of rheumatoid arthritis (RA). Investigation to identify predictive tools of bDMARD response is a highly active and prolific area of research. In addition to clinical phenotyping, cellular and molecular characterisation of synovial tissue and blood in patients with RA, using different technologies, can facilitate predictive testing. This narrative review will summarise the literature for the available bDMARD classes and focus on where progress has been made. We will also look ahead and consider the increasing use of ‘omics’ technologies, the potential they hold as well as the challenges, and what is needed in the future to fully realise our ambition of personalised bDMARD treatment.

## Background

The management of rheumatoid arthritis (RA) has been transformed with the advent of biologic disease-modifying anti-rheumatic drugs (bDMARDs) targeting key cells and molecules of disease pathophysiology [1, 2]. However, a lack of universal response is seen with currently available therapy [1]. As such, even in this exciting therapeutic era the vast majority of patients fail to achieve the desired high-level response (equivalent to low disease activity or remission), and almost 40% of all patients treated with bDMARDs do not even experience minimally acceptable improvement. This means individual patients are treated sequentially with different drugs, selected using little mechanistic rationale; consequently leading to increased costs, unnecessary toxicity and suboptimal effectiveness [3]. Furthermore, the varied response pattern reflects the increasingly recognised concept of RA as a *syndrome*—that is, heterogeneous aetiology and pathophysiology with many immunological variants and a common clinical phenotype [4]. The cumulative evidence base highlights the existence of several pathobiological signatures that may associate with individual immunopathogenic patient profiles, and thus a rational specific therapeutic agent [5–8]. Thus, it is somewhat expected that the same treatment strategy will not achieve similar results in every RA patient.

With this in mind, an overarching ambition has emerged to deliver targeted therapies according to the individual patient profile and disease endotype. This core principle of personalised medicine has driven considerable efforts in the identification of response predictors, both clinical and biological.

Of the significant number of studies exploring response prediction, this narrative review will summarise the mainly biological investigations considered to be of particular relevance and interest, focusing on currently available bDMARDs. Looking ahead, we will comment on the emerging role of multi-omics approaches (genomics, transcriptomics, proteomics, metabolomics) as well as the challenges they pose.

## Generic clinical predictors of response

A number of generic clinical predictors of response to most classes of bDMARD therapy have been reported. Concurrent treatment with DMARDs, specifically methotrexate (MTX), is one of the most significant predictors of response to bDMARD therapy and all biologics are recommended to be administered in combination therapy [1, 9, 10]. This effect has been suggested to be related to modulation of bDMARD immunogenicity, through limitation of neutralising anti-drug antibody development that can lead to reduced serum drug levels and treatment failure [11]. However, MTX may also improve response by inhibiting other more diverse immune pathways in addition to those targeted by a biologic. Current smoking is associated with worse response to tumour necrosis factor inhibitors (TNFi) and is the most important modifiable variable [12–16]. Other identified predictors mainly include markers of disease severity that predict poor therapeutic outcome, thus not enabling treatment individualisation. Lower disability as assessed by the health assessment questionnaire (HAQ) and higher baseline 28-joint disease activity score (DAS28) is associated with better response to bDMARDs—the latter when American College of Rheumatology (ACR) response is considered [9, 10, 14, 17]. Other factors associated with good response include male sex, younger age, early disease and lower number of previous bDMARDs [18, 19].

## Biological response predictors across biologic agents

### Serological status

Presence of rheumatoid factor (RF) and anti-citrullinated protein antibodies (ACPA) is currently the only applicable means of treatment stratification at the disease level. There is grade Ia evidence [20] supporting a clearly better response for rituximab, an anti-CD20 monoclonal antibody, in seropositive patients (for RF and/or ACPA), including a significant effect at the joint damage level [21]; with this association confirmed in large observational cohort studies [22, 23]. Antibody status predicted response best in the TNFi-resistant cohort, while in therapy-naïve patients the predictive effect of antibody status tended to depend on the presence of clinical markers of severity of inflammation, indicating the need to include clinical predictors in biomarker analyses. In contrast, registry data suggest that TNFi may perform worse in seropositive patients, particularly in RF-positive patients [14, 17, 19, 24]. However, this finding has not been validated in clinical trials and a recent systematic review and meta-analysis concluded that both RF and ACPA status were not predictive of response to TNFi [25]. With abatacept, despite a negative meta-analysis published in 2013 that did not find an association between RF and clinical response [23], a very recent study provided a pooled analysis of nine European registries including over 2700 patients, reporting that both ACPA and RF positivity were associated with reduced likelihood of abatacept discontinuation for ineffectiveness or any reason [26]. The same meta-analysis from 2013 [23] analysed results from five studies of tocilizumab (*n* = 1844) and reported a significantly better ACR20 response for RF-positive patients (odds ratio (OR) 1.51, 95% confidence interval (CI) 1.21–1.90, *I* ^2^ = 0.0%). However, the specific relationship of interleukin (IL)-6 and C-reactive protein (CRP) that informs ACR and European League Against Rheumatism (EULAR) response scores is particularly relevant when evaluating tocilizumab; studies which use measures that do not include CRP, such as the clinical disease activity index (CDAI), would overcome such confounding factors. A small study with 58 patients found high RF titres to be associated with CDAI remission at 24 weeks [27]. In three other more recent studies, however, RF status did not associate with EULAR response at 24 weeks (*n* = 204) [28] or with CDAI (*n* = 102) [29] or CDAI remission (*n* = 839) [30] at 52 weeks.

### Myeloid-related proteins

A generic biomarker of response that has emerged in recent years is the protein complex of myeloid-related proteins (MRP) 8/14, also known as calprotectin. MRP are enrolled in the myeloid (i.e. monocyte/macrophage) inflammatory component of synovitis and their systemic levels have been shown to strongly correlate with clinical (for DAS28 *r* = 0.89, *p* < 0.001) and ultrasound (*r* = 0.64, *p* < 0.001) disease activity [31–33]. Moreover, higher MRP8/14 baseline levels have been associated with EULAR response to adalimumab (OR 3.14, *p* = 0.04; area under the curve (AUC) 0.688), infliximab (OR 7.82, *p* = 0.006; AUC 0.791) and rituximab (OR 210.21, *p* = 0.002; AUC 0.984), after adjustment for baseline DAS28 and 68-joint tender joint count (the only two other significant variables on univariate analysis), and a consistent decrease was seen in this marker, parallel to clinical improvement [32, 34]. The same authors recently applied these results to development of a treatment algorithm that used a prediction score including MRP8/14 baseline serum levels and generic clinical variables (baseline DAS28 and HAQ, RF positivity, drug class (rituximab vs TNFi) and previous TNFi use) [19]. In 59% of patients a recommendation on treatment class (TNFi, rituximab, other drug class) could be made and the predicted probability of response with this model matched the observed response in the cohort very well, with only 10% difference between the model and the cohort in patients who followed recommendations and a clearly larger difference in those who were treated contrary to the algorithm [19]. It should be noted, however, that this algorithm was derived and tested in a single cohort of 170 patients and that the other clinical variables also largely contribute to adequately distinguish therapy-specific response.

### Type I interferons

Type I interferon (IFN) activity has also been associated with response to biologics in a differential manner. However, studies have been modestly sized, with different approaches employed to measure IFN activity. Since there are numerous subtypes of type I IFN, which are difficult to detect in serum, expression of a selection of interferon-stimulated genes (ISGs) is often used instead, although these may not exclusively respond to type I IFN. Upregulation of a cluster of ISGs on a micro-array may be referred to as an IFN signature.

Despite the differences in the exact genes measured, the presence of an IFN signature, or micro-arrays in whole blood or synovial tissue, as well as raised expression of three ISGs using quantitative polymerase chain reaction (qPCR) predicted poor response to rituximab in three independent studies [35–37]. Interestingly, an IFN-high profile was associated with increased response to TNFi in two studies; one measured ISG expression in neutrophils while another measured serum IFN-I activity using a reporter cell assay [38, 39]. Studies that used blood micro-arrays have not yet demonstrated an association between baseline IFN signature and clinical response [40, 41]. Blood IFN signature and qPCR ISG expression have been associated with better response to tocilizumab [42]. In several of these studies, the expression of ISGs at baseline was associated with inflammatory markers or DAS28. Thus, available evidence suggests that patients with an IFN signature may have greater benefit from tocilizumab than rituximab, but due to variation in assays and the need to adjust for other clinical characteristics this remains uncertain. In addition, the role of IFN in RA may be more complex. IFN-α is predominantly produced by circulating plasmacytoid dendritic cells, and is generally associated with worse outcomes in autoimmune diseases [43]. In contrast, local tissues including synovial fibroblasts produce more IFN-β. Data from animal [44–46] and human [47–49] studies suggest IFN-β may have more of a regulatory role, being associated with lower levels of TNF and higher levels of transforming growth factor (TGF) beta, IL-10 and IL-1RA. In systemic lupus erythematosus, all plasma IFN activity was attributable to IFN-α, while IFN-β also contributed to the IFN profile in RA.

## Biological response predictors for specific biologics

Other biomarkers have been investigated to predict response to specific bDMARD classes. Rather than fully characterising predominant pathogenic processes, they are often related to the mechanism of action of a given drug and may estimate the probability of response.

### TNF inhibitors

A wide range of genetic, epigenetic and gene expression studies assessing key players of the inflammatory response have emerged recently, raising exciting hypotheses but still failing to consistently differentiate responders from non-responders across different TNFi-treated RA cohorts.

#### Genome-wide association studies

Genome-wide association studies (GWAS) have identified a number of different loci associated with TNFi response in Caucasian and Asian populations, but other large independent studies have failed to confirm these associations [5, 6, 50–54]. Moreover, only two loci have reached genome-wide significance: PDE3A-SLCO1C1, containing genes encoding a phosphodiesterase A and a member of the anion transporter family, for which the C > T polymorphism was associated with reduced efficacy to adalimumab, etanercept and infliximab with high significance (*p* = 10^–11^) [53, 55]; and CD84, encoding SLAM family member 5, in which the G > A single nucleotide polymorphism (SNP) predicted responsiveness to etanercept (*p* = 10^–8^) [56]. The predictive value of these SNPs has thus far not been confirmed and is insufficient in selecting individual treatment selection. Indeed, a more recent study with a sample size five to nine times larger than previous ones failed to replicate the association of PDE3A-SLCO1C1 with response to TNFi [54]. Other SNPs have been associated with response to TNFi in hypothesis-driven studies with large numbers of patients (reviewed in detail elsewhere [5, 6, 50]). These include conflicting data on G308A SNP at the TNF gene (308GG genotype linked to better response to TNFi in two meta-analysis but refuted in another, larger and robust one [5]); and rs10919563 G > A SNP at the RA susceptibility-associated gene PTPRC (G allele associated with good response to TNFi in at least large three studies but not in another similarly large cohort and meta-analysis) [57–60]. Such inconsistency and/or only explaining a small amount of the observed response mean genetic factors remain insufficient to be applied at the clinical level. This notion was confirmed in a very recent important study which used collective SNP data from dozens of research groups and concluded that SNP information did not add significant value to standard clinical variables and that the research focus should be re-centred elsewhere [61].

#### Transcriptomic studies

Alternative approaches such as gene expression analysis have thus emerged. Transcriptomics is a high-throughput technique that studies the whole RNA signature of a given cell or tissue in a specific time. Despite being more prone to variation due to other intrinsic or extrinsic factors, it is very robust and has high discriminatory power even in small cohorts [50, 62, 63]. Several gene expression signatures associating with response to TNFi have been identified, but few replicated, and except for the type I IFN signature mentioned previously, no other clear signals are evident. A multiplicity of reasons may explain the inconsistencies, including study design (heterogeneous and small cohorts, different disease stages or time points considered and the analysis of distinct tissues or even cell types) and technical/analytic approaches (different transcriptomic platforms, a high false positive rate due to multiple testing, different computational analysis methods). Indeed, important cell-type specificities have been reported and can be missed when whole tissue (e.g. blood or synovium) is tested [62]. Nonetheless, transcriptomics still has tremendous potential in the field of personalised medicine, with the investigation of synovial tissue, the primary site of disease, offering particular promise. The advent of minimally invasive techniques such as ultrasound-guided synovial needle biopsies (USNB) [64] facilitates necessary access to the main site of inflammation [62]. At the moment though, transcriptomics is not yet ready for the prime time, and more well-designed, uniform, powered studies are needed in order to replicate results and establish clear signals that can be applied in clinical practice.

#### Epigenetics

Epigenetic changes (e.g. DNA methylation) control gene expression and might influence disease risk, prognosis and eventually drug response [6, 65]. Epigenetic regulation of key inflammatory genes (e.g. TNF) could in theory influence response to TNFi [6, 50]. Several epigenome-wide studies have been conducted, and are ongoing in the search for a discriminative DNA methylation signature that predicts response to TNFi (comprehensively reviewed in detail elsewhere [6]). Non-coding microRNAs also modulate gene expression through repression of DNA translation, and deregulation of a number of them has been identified in RA, both at the tissue and systemic levels [66]. A few recent studies have investigated the role of non-coding microRNAs as potential predictors of response to TNFi and identified several microRNAs with good discriminative ability, but only one (microRNA 23) was replicated in separate studies [67–69]. Interestingly, one study reported distinct signatures for different TNFi, namely etanercept and adalimumab, suggesting that microRNA regulation may differ according to TNFi type (monoclonal antibody vs fusion receptor protein) and, thus, studies grouping different TNFi together can hamper detection of a predictive signal and the conclusions [68].

#### Synovial tissue

Different synovitis patterns have been described at the cellular and molecular levels, defined by clusters of genes related to the myeloid or lymphoid (B-cell-related) inflammatory compartments, or even associated with fibroblast and bone turnover processes (fibroid) [70]. As such, synovial tissue analysis is likely to be of key importance in the field of personalised medicine. The role of CD68^+^ sub-lining macrophages as (a generic) marker of response to DMARDs is well established [71]. Increased myeloid synovial infiltration and higher synovial expression of TNF and other macrophage-related inflammatory genes have been associated with response to infliximab, although only explaining a small part of response variation [70, 72, 73]. Moreover, a synovium gene signature closer to the lymphoid inflammatory pathways (including ectopic lymphoid neogenesis (ELN)) did not associate with response to infliximab [74], and consistent with this another study reported that a serum lymphoid synovial signature (low serum levels of soluble intercellular adhesion molecule 1 (sICAM1)/high serum levels of CXCL13) was associated with poor response to adalimumab (ACR50 13%) [70]. These results seem to suggest that an overall predominance of the TNF pathway and myeloid infiltration at the tissue level lead to better response to TNFi. In line with this, baseline synovium lymphoid infiltration with the presence of ELN has been negatively associated with response to TNFi in a landmark study with 86 biopsied patients [75], as were increased synovial fluid IL-6 levels [76]. However, another key study with 97 patients reported contradicting findings, with synovial ELN being associated with better response to infliximab [77]. Methodological and technical reasons in addition to clinical and treatment variables might explain these differences. Importantly, adding the presence of synovial lymphoid aggregates to the prior model with clinical variables and TNF expression increased the performance of the model from 19 to 29% of variation of response, which is clearly insufficient to be used as a predictive test in clinical practice. Overall, synovial inflammatory pathway analysis bears great potential and the dissemination of techniques like USNB may facilitate access to tissue and enable more hypothesis-driven studies and/or confirm the data so far available. Currently, tissue-based treatment personalisation remains elusive.

#### Serum markers

Peripheral blood and serum have frequently been employed in the search for biomarkers of response. As mentioned earlier, the translation of biomarkers that best characterised lymphoid and myeloid synovial phenotypes at the gene level (CXCL13 and ICAM1, respectively) into their serum surrogates was able to differentiate response to adalimumab (ACR50 response of 42% if sICAM^high^/CXCL13^low^) and tocilizumab (ACR50 of 69% if sICAM1^low^/CXCL13^high^) in a different cohort of patients [70]. However, it should be noted that there were no synovial tissue data available for the patients evaluated for response, and in another study that analysed paired synovial tissue and peripheral blood samples the authors did not find a differential gene expression in the blood that matched the high/low inflammatory profile exhibited at the tissue level [78].

While pre-treatment TNF blood levels or mRNA expression have been shown not to correlate with response to TNFi [76, 79–81], higher circulating TNF bioactivity assessed through in*-*vitro induction of TNF-related cytokines (IL-1β, IL-6) was associated with better treatment response to TNFi in three small studies [81–83]. Also, a recent study used in-vitro testing of peripheral blood monocytes of patients treated with TNFi to identify that transmembrane TNF crosslinking induced concentrations of soluble TNF receptor 1, soluble IL-1 receptor 1 and IL-10 strongly associated with good EULAR response (AUC 0.91–1.00) [84]. Consistent with the hypothesis that predominance of non-TNF pathways may lead to TNFi resistance, increased IL-17 levels and Th17 cell frequency were associated with poor response to TNFi [81, 85].

Considering the importance of TNF in cartilage–bone turnover and joint destruction, serum biomarkers such as matrix metalloproteinase 3 (MMP3) have been investigated and associated with response to infliximab [86]. This finding was not confirmed in other studies assessing a wide variety of bone/cartilage-related markers as predictors of response to TNFi [87, 88]. Lower levels of cartilage oligomeric matrix protein (related to cartilage turnover) and receptor activator for nuclear factor-κB ligand (RANKL) and lower RANKL:osteoprotegerin ratio (both associated with bone reabsorption) have all been associated with better response to adalimumab or infliximab [89, 90].

##### Metabolomics

The characterisation of metabolites in a given system has also been employed as a tool to predict response to TNFi and three recent studies identified baseline serum/urine metabolite signatures associated with clinical response to TNFi with good accuracy [91–93]. More studies are needed to confirm these results, which are nevertheless very encouraging. Finally, Table 1 summarises findings from proteomic studies that used this technology to identify protein signatures able to predict response to TNFi with good to excellent accuracy. Again, few consistent signals have emerged to date.

**Table 1:** Summary of proteomic studies investigating response to biologic therapy

Biomarker	Sample size	Treatment	Main results	Reference
24 autoantibodies and cytokines^a^	3 independent cohorts, *n* = 93	ETN	PPV 58–72% NPV 63–78%	Hueber et al. [150]
7 proteins including acute phase reactants, proteins of the complement system^b^	2 independent cohorts, *n* = 22/16	ETN	AUC R/NR 0.86–1.0 PROS and CO7: sens 88.9%, spec 100%	Obry et al. [151]
12 cytokines and chemokines^c^	*n* = 33	ETN	MCP1, EGF: good response CRP + EGF: good response (AUC 0.844, sens 87.5%, spec 75%)	Fabre et al. [80]
14 proteins enriched in apolipoproteins, components of the complement system and acute phase reactants^d^	*n* = 8	IFX	NR/R ratio 1.336–5.459 AUC 0.875–1.0	Ortea et al. [152]
6 proteins signalled, 2 identified: apolipoprotein A and platelet factor 4	*n* = 60	IFX	AUC for all proteins 0.761–0.846 Combination: sens 97.1%, spec 97.5% Apo-A: good response, PF4: non-response	Trocmé et al. [153]
12 biomarkers assembled into one multi-biomarker disease activity (MBDA) score	*n* = 144	IFX vs triple tx^f^	Rapid radiographic progression lower with IFX if high MBDA	Hambardzumyan et al. [154]
9 proteins differentiated response^e^	*n* = 8	ADA	NR/R 1.42–2.18/0.42–0.73. Independence to IFX results	Ortea et al. [155]
12 cytokines and chemokines^c^	*n* = 46	RTX	Baseline cytokines profiles not related to response	Fabre et al. [124]

Original table summarising proteomic studies available to date that aimed to investigate response to biologic therapy in rheumatoid arthritis

*ADA* adalimumab, *AUC* area under the curve, *ETN* etanercept, *IFX* infliximab, *NPV* negative predictive value, *NR* non-responder, *PPV* positive predictive value, *R* responder, *sens* sensitivity, *spec* specificity, *RTX* rituximab, *tx* therapy

^a^GM-CSF, interleukin (IL)-6, fibromodulin, clusterin, ApoE, H2B/e, clusterin, HSP58, IL-1α, COMP, acetyl-calpastatin, biglycan, osteoglycin, serine protease-11, IL-1β, eotaxin, IP-10, FGF-2, MCP-1, IL-12p70, fibrinogen, FibA, IL-12p40, IL-15

^b^Ceruloplasmin, complement component C7 (CO7), inter-alpha-trypsin inhibitor heavy chain 1, plasminogen, vitamin K-dependent protein S (PROS), protein S100A9, zinc-alpha2-glycoprotein

^c^IL-6, TNF-α, IL-1α, IL-1β, IL-2, IL-8, IFN-γ, IL-4, IL-10, monocyte chemoattractant protein (MCP)-1, epidermal growth factor (EGF), vascular endothelium growth factor

^d^Vitamin D-binding protein splicing variant GC-006, ceruloplasmin, apolipoprotein B-100, inter-alpha-trypsin inhibitor heavy chain H2, thrombospondin-1, complement C4-B alpha chain, inter-alpha-trypsin inhibitor heavy chain H1, gelsolin, apolipoprotein A-II, fibronectin isoform 7, complement factor H-related protein 4, apolipoprotein M, adipocyte plasma membrane-associated protein, mannan-binding lectin serine protease 2

^e^Tropomyosin alpha-4 chain, Transgelin-2, Cofilin-1, Hemopexin, complement C3, SH3 domain-binding glutamic acid-rich-like 3, transcription factor-like 5 protein, target of Nesh-SH3, Isoform 2 of Tropomyosin alpha-3 chain

^f^Triple disease-modifying anti-rheumatic therapy: methotrexate, hydroxychloroquine, sulfasalazine

### Rituximab

#### B-cell phenotyping

Rituximab depletes CD20-positive B cells. There has therefore been a focus on enumeration of B-lineage cells in blood and synovium as predictive biomarkers, as well as other markers of B-cell function, such as secreted immunoglobulin and B-cell cytokines. Prior experience using cell-depleting therapies in haematology has demonstrated the value of measuring the extent of B-cell depletion as a biomarker.

In addition to autoantibodies, markers of B-cell activity may also predict better clinical response, such as raised serum IgG, the B-cell cytokine BAFF or the chemokine CCL19 [94–96]. In contrast, in the synovium, higher numbers of CD79a^+^ B cells at baseline predict worse clinical response [97]. In the blood, three studies have used flow cytometry to demonstrate that higher numbers of circulating plasmablasts predict worse clinical response [98–100]. This has been confirmed using a large cohort of patients pooled from randomised trials using a plasmablast gene expression signature based on the combination of IgJ and FCRL5 mRNA expression that predicted non-response to rituximab [101].

Plasmablasts are a plasma cell precursor differentiated from activated B cells. They are short-lived in the circulation and are CD20 negative, so may act as a biomarker of B-cell activity, especially after depletion of CD20-positive B cells. However, they are not detected in a CD19 lymphocyte gate, requiring specialised flow cytometry protocols for accurate enumeration after rituximab, called high-sensitivity flow cytometry.

Complete depletion of plasmablasts after the first infusion, assessed through high-sensitivity flow cytometry, has been clearly associated with better clinical outcomes, compared with non-complete depletion [102]. Plasmablast levels may also explain the more variable response to lower dose rituximab: although the rate of complete depletion was lower with lower dose rituximab, patients with lower baseline plasmablasts counts could achieve complete depletion and good EULAR response. Moreover, for patients who failed to deplete, a third extra dose of rituximab increased complete depletion rates and this was associated with better clinical response [103]. These data provide a basis for modifying therapy. However, studies that used different flow cytometric protocols did not reproduce these findings [104, 105]. Another study that used high-sensitivity flow cytometry reproduced baseline, but not depletion, results [100].

Clinical responders have also been found to have lower baseline frequency, more profound suppression and delayed resurgence of memory B cells [106–109]. Also, an increased number of plasma CD95^+^ activated B cells and class-switched memory B cells at depletion, and a lower transitional-to-memory B-cell ratio at reconstitution were associated with poor response; class-switched memory B cells accumulated in flaring joints, confirming the pathogenic role of these cells in RA [110, 111]. Clinical relapse is usually preceded a few months by B-cell compartment repopulation and memory B cells seem to be key players in this process [107, 112].

Synovial tissue data underline the variable B-cell response to standard-dose rituximab that was demonstrated in blood. Depletion of synovial B cells is more variable. This is less clearly related to treatment response, although these studies have been very modest in size [97, 105, 113, 114]. In one synovial study, greater local B-cell depletion (assessed through CD19 mRNA expression but not through histology) was seen in ACR50 responders (but not overall responders) compared with non-responders and was coupled with decreased synovial immunoglobulin production [105, 115].

Greater decrease of synovial plasma cells was reported in good responders (*R* ^2^ = 0.26), correlating with the reduction in serum ACPA levels [113]. Lymphoid aggregates have been found to decrease after 16 but not 4 weeks. However, baseline synovial plasma cells and lymphoid aggregates did not predict treatment outcome and it is therefore less clear whether the greater normalisation of these changes in clinical responders is a rituximab-specific mechanism of response or just another (generic) reflection of an overall improvement in synovitis [105, 113]. Interestingly, type I IFN has a key role in promoting the differentiation of plasmablasts and plasma cells from B cells, which may link the negative predictive value of the IFN signature with these blood and synovial findings [116]. Overall, synovial cellular markers have provided clues to rituximab’s mode of action and RA pathogenesis, but have been limited in associating with clinical outcomes. Large multicentre tissue-based randomised clinical trials (RCTs) further investigating the role of synovium B-cell burden in predicting response to rituximab are ongoing [117].

#### Transcriptomic studies

Gene expression studies have emerged, building on evidence from cellular-focused blood and tissue research [62, 63]. The clearest signal comes from the already mentioned type I IFN signature, negatively associated with response to rituximab at the whole blood and peripheral blood mononuclear cell (PBMC) levels [35, 36, 118]. Besides baseline expression, the induction of type I IFN response genes 3 months after rituximab treatment was also associated with good clinical response at 6 months [118]. An important study assessed 68 patients from the SMART study and found a whole-blood transcriptomic signature associated with 6-month response to rituximab, including upregulation of the NF-κB pathway and downregulation of the IFN pathway, which correctly classified treatment response in 92.6% of cases [119]. This was also confirmed at the tissue level, where patients with a high inflammatory gene score, overexpressing macrophage and T-cell-related genes and under-expressing IFN and remodelling genes, responded better to rituximab [37]. Also in line with this, another study found responders to have upregulation of synovium immunoglobulin genes and of genes involved in antigen processing and MHC class II presentation [120].

Investigation into other biomarker candidates is more limited for rituximab response prediction than for TNFi. Some markers have been associated with better response, including: SNPs of the Fc gamma receptor 3A (158 V > F, VV genotype) [121], BAFF (871C > T, C allele carriage) [122] and IL-6 (174G > C, CC genotype) [121] genes; micro-RNA-125b (increased expression) [123]; and cytokine profile assessed through proteomic analysis (Table 1) [124].

### Abatacept

Abatacept is a soluble fusion protein of the modified Fc region of human IgG1 and the extracellular domain of cytotoxic T-lymphocyte-associated antigen 4 (CTLA-4) which binds to the CD80/CD86 complex, modulating CD28-mediated T-cell activation [125]. Except for ACPA/RF positivity (see earlier), there are currently no consistent biomarkers of response validated in separate cohorts. However, a few studies have provided some mechanistic insights into the mode of action of abatacept, with modulation of not only T cells but also B-cell biology observed: abatacept significantly decreases synovial B-cell infiltration and gene expression of IFN-γ, IL-1β, MMP1 and MMP3 (which was greater in responders) [126]; it leads to reductions of serum immunoglobulins and free light chains, ACPA/RF titres and post-switch memory B cells [127], as well as effector (Th1, Th2, Th17) and regulatory T cells (Tregs) [128] and follicular helper T cells, the latter being related to the inhibition of Syk phosphorylation in B cells seen with abatacept [129]. Another important study also found in TNFi inadequate responder (IR) patients that abatacept restored B-cell proliferation, plasma cell differentiation and modulatory proprieties of regulatory T cells, which were all impaired before therapy, and this was related to clinical improvement [130].

The most instructive baseline predictive marker to date appears to be the blood count of immunosenescence-associated CD28^–^ T-cell count. Lower baseline levels of CD4^+^CD28^–^ cells (<28/μl) and, especially, CD8^+^CD28^–^ T cells (<87/μl) strongly predicted remission at 6 months (hazard ratio 3.3 and 4.4, respectively, *p* < 0.001) [131]. CD28^–^ T cells have functional characteristics of cytotoxic cells (such as NK cells) and have been proposed to play a role in the pathogenesis of RA [132]. Interestingly, in a more recent study assessing pre-treatment whole blood gene expression, a NK-cell-related signature was associated with poor response to abatacept with good accuracy (AUC 0.768) [133], suggesting a replication of flow cytometry results for CD28^–^ T cells as markers of abatacept failure. A greater decrease in serum levels of A disintegrin and metalloprotease 17 (ADAM17), a cleaving enzyme responsible for shedding of TNF and other cytokines, has also been observed in responsive patients [134].

### Tocilizumab

Tocilizumab blocks the IL-6 receptor (IL-6R) and it is not surprising that most prediction studies have focused on the IL-6 pathway, key to RA pathogenesis. Genetic polymorphisms have also been studied as biomarkers of response to tocilizumab. While no IL-6 or IL-6R SNPs were found to be significantly associated with tocilizumab treatment outcomes [135, 136], a recent GWAS study identified eight loci, none of which were previously linked with RA, drug response, the IL-6 pathway or the shared epitope [137]. However, a recent small study studied the significance of these SNPs and confirmed two of them (GALNT18 rs4910008, C > T and CD69 rs11052877, A > G) as positively associated with response (C-allele carriers and A-allele carriers, respectively) [138].

Recent genomic studies have tried to look for systemic and local biomarkers of response to IL-6R blockade. Genome-wide analysis of PBMCs identified upregulation of three type I IFN response genes (IFI6, MX2, OASL) and one gene encoding metallothionein-1G (the promoter of which is upregulated by IL-6) in tocilizumab good/moderate responders (best AUC of 0.947 with two gene combinations) [42]. Another study did not mention the IFN signature but identified increases in the expression of *TRAV8-3* (involved in CD8 T-cell response), *EPHA4* and *CCDC32* and a decrease for *DHFR* (dihydrofolate reductase, associated with response to MTX) in PBMCs of patients responding to tocilizumab [139]. These authors also reported increased IgG glycosylation in association with response, a finding that lacks confirmation. Whole blood mRNA expression of IL-6R was also not associated with response to tocilizumab, confirming findings for serum levels of this molecule [136]. At a tissue level, tocilizumab was found to significantly decrease lymphoplasmacytic cell infiltrates as a whole and individual counts of macrophage, T cells and plasma cells (but only a trend for B cells) as well as expression of IL-7, CCL2, CXCL13 and CCL8 [140]. There was no difference in this pattern of change or baseline histological features according to remission status at 6 months, but overexpression of genes involved in Ras protein signal transduction and cell cycle pathways was seen in responders. Importantly, tocilizumab seemed to induce molecular changes similar to rituximab and methotrexate but different to those seen with adalimumab. In line with this, enrichment of TNF-induced gene transcripts in synovial samples of early RA patients was associated with poor response to tocilizumab [141], and in the previously mentioned study by Dennis et al. [70] a serological cytokine signature (sICAM1^high^/CXCL13^low^) that correlated with myeloid TNF-rich mechanisms at the synovial level was negatively associated with the ACR50 response to tocilizumab (20%), whereas the opposite profile, surrogate of a lymphoid synovial signature (sICAM1^low^/CXCL13^high^), strongly predicted clinical response to IL-6R blockade (ACR50 69%).

Baseline serum IL-6 (but not IL-6R) levels have been associated with response to tocilizumab, but with contradicting results [136, 142–144]. Both low [142, 143] and high [136, 144] IL-6 levels were proposed as markers of good response. However, even considering that persistently high IL-6 levels in patients failing other biologics like rituximab may suggest a predominance of IL-6-related pathways and a likely better response to tocilizumab [144], the overall clinical effect of baseline IL-6 levels is probably limited (especially in TNFi IRs) as they were ineffective in discriminating responders from non-responders in a large pool of patients (AUC 0.59) [136]. The various elements involved in IL-6 signalling, and the impact of the relative expression of IL-6 ligand, soluble IL-6R, on classic and trans signalling of IL-6 makes interpretation of singular markers challenging. Indeed, a recent study assessed 31 cytokines/chemokines/soluble receptors and found that the combination of soluble gp130Fc, IL-6, IFN-γ-induced protein 10 and soluble TNF receptor II strongly predicted DAS28 remission after tocilizumab therapy (AUC 0.85/0.89 for naïve/non-naïve patients) [143]. Soluble gp130Fc, a natural antagonist of IL-6/IL-6R, was the most robust positive predictor of response (AUC 0.74–0.81). Low IL-17A levels have also been linked with higher remission rates, but estimation of the effect and replication of this finding are missing [145]. A few cellular markers of response to tocilizumab have been suggested, including lower baseline frequency of CD27^–^IgD^–^ B cells [146] and greater increase in the proportion of Tregs among CD4^+^ T cells after treatment start [147].

## Summary

bDMARD response biomarker research in RA has been a trending area for over a decade, but few consistent signals aside from serological status have emerged for implementation into clinical practice. Whilst available data may guide treatment decisions to a degree (perhaps mainly supporting TNFi over other therapies in a seronegative patient), there are limitations in being able to refine decisions between targeted therapies. The IFN signature seems promising but a number of questions remain unclear. With several markers mainly reflective of generic markers of disease severity and of responsiveness to therapy, there is a need to identify therapy-specific predictive markers. In addition, consistency in approach is needed to mitigate against conflicting data driven by varied patient populations studied, time points evaluated, definition and accuracy of response definition, tissue/cell populations studied and methods employed. Finally, whilst the plethora of studies provide insights into RA pathophysiology and drug response mechanisms, potentially valuable signals are not fully progressed along the translational pathway, towards stratified clinical studies that are necessary to deliver clinical meaningfulness and impact.

## Future direction

Medical fields, for example oncology, have pursued response signatures more successfully, most prominently observed in breast cancer, with oestrogen receptor or HER2 positive biopsy predictive of good response to tamoxifen or trastuzumab, respectively [62]. Nevertheless, it should be noted that even in these cases a satisfactory response is observed in only approximately 50% of patients, with intra-tumour heterogeneity accounting for the gap in complete response association [148]. As well as managing our expectations, these observations underscore the importance of studying pathogenic mechanisms at the primary site of disease. Whether a tissue-based biomarker in systemic inflammatory diseases such as RA is as crucial compared with tumour biology and is of sole importance, and whether this is dependent on therapy class, remains unclear. Evaluation of biology at both the systemic and local tissue levels is likely to be relevant in heterogeneous diseases such as RA.

With this in mind, high-throughput omics techniques (genomics, transcriptomics, proteomics, metabolomics) are increasingly being employed. A number of challenges exist, including the risk of more spurious associations that whilst perhaps statistically significant and biologically plausible have little or no clinical impact. Integrative multi-omics approaches aim to overcome this [149], with, in parallel, improved analytical methods to increase the detection of accurate response predictors. Moreover, collaborative initiatives and consortia should help address discrepant findings [61], support more uniform approaches to experimental assays and study designs, and overcome the limitations of piecemeal studies or the lack of head-to-head populations to compare biomarkers.

## Conclusions

Individualisation of bDMARD therapy in RA is not yet a reality, but encouraging data gathered over the last decade together with the emergence of powerful techniques, and the continued investment in this area, will hopefully lead to the identification of novel biomarkers that can optimise treatment selection in clinical practice and improve patient outcomes. Until then clinicians will have to follow current treatment strategies, integrating limited generic predictors with other patient, drug and social/economic factors when choosing from the available therapies.

## Abbreviations

ACPA: Anti-citrullinated protein antibodies
ACR: American College of Rheumatology
ADAM17: A disintegrin and metalloprotease 17
AUC: Area under the curve
bDMARD: Biological disease-modifying anti-rheumatic drug
CDAI: Clinical disease activity index
CI: Confidence interval
DAS28: 28-Joint disease activity score
DMARD: Disease-modifying anti-rheumatic drug
ELN: Ectopic lymphoid neogenesis
EULAR: European League Against Rheumatism
GWAS: Genome-wide association study
HAQ: Health assessment questionnaire
IFN: Interferon
IL: Interleukin
IL-6R: Interleukin-6 receptor
ISG: Interferon-stimulated gene
MMP: Matrix metalloproteinase
MRP: Myeloid-related proteins
MTX: Methotrexate
OR: Odds ratio
PBMC: Peripheral blood mononuclear cell
qPCR: Quantitative polymerase chain reaction
RA: Rheumatoid arthritis
RANKL: Receptor activator for nuclear factor-κB ligand
RCT: Randomised clinical trial
RF: Rheumatoid factor
sICAM1: Soluble intercellular adhesion molecule 1
SNP: Single nucleotide polymorphism
TNFi: Tumour necrosis factor inhibitors
USNB: Ultrasound-guided synovial needle biopsies

## References

[1] Nam JL, Winthrop KL, van Vollenhoven RF, Pavelka K, Valesini G, Hensor EM (2010). Current evidence for the management of rheumatoid arthritis with biological disease-modifying antirheumatic drugs: a systematic literature review informing the EULAR recommendations for the management of RA. Ann Rheum Dis..

[2] van Nies JAB, de Jong Z, van der Helm-van Mil AHM, Knevel R, Le Cessie S, Huizinga TWJ (2010). Improved treatment strategies reduce the increased mortality risk in early RA patients. Rheumatology..

[3] Joensuu JT, Huoponen S, Aaltonen KJ, Konttinen YT, Nordström D, Blom M (2015). The cost-effectiveness of biologics for the treatment of rheumatoid arthritis: a systematic review. PLoS One..

[4] Firestein GS (2014). The disease formerly known as rheumatoid arthritis. Arthritis Res Ther..

[5] Gibson DS, Bustard MJ, McGeough CM, Murray HA, Crockard MA, McDowell A (2015). Current and future trends in biomarker discovery and development of companion diagnostics for arthritis. Expert Rev Mol Diagn..

[6] Plant D, Wilson AG, Barton A (2014). Genetic and epigenetic predictors of responsiveness to treatment in RA. Nat Rev Rheumatol..

[7] Dennis G, Holweg CT, Kummerfeld SK, Choy DF, Setiadi AF, Hackney JA (2014). Synovial phenotypes in rheumatoid arthritis correlate with response to biologic therapeutics. Arthritis Res Ther..

[8] Townsend MJ (2014). Molecular and cellular heterogeneity in the Rheumatoid Arthritis synovium: Clinical correlates of synovitis. Best Pract Res Clin Rheumatol..

[9] Hyrich KL, Watson KD, Silman AJ, Symmons DPM, Register TBB (2006). Predictors of response to anti-TNF-alpha therapy among patients with rheumatoid arthritis: results from the British Society for Rheumatology Biologics Register. Rheumatology (Oxford).

[10] Kristensen LE, Kapetanovic MC, Gülfe A, Söderlin M, Saxne T, Geborek P (2008). Predictors of response to anti-TNF therapy according to ACR and EULAR criteria in patients with established RA: results from the South Swedish Arthritis Treatment Group Register. Rheumatology (Oxford).

[11] van Schouwenburg PA, Rispens T, Wolbink GJ (2013). Immunogenicity of anti-TNF biologic therapies for rheumatoid arthritis. Nat Rev Rheumatol..

[12] Saevarsdottir S, Wedrén S, Seddighzadeh M, Bengtsson C, Wesley A, Lindblad S (2011). Patients with early rheumatoid arthritis who smoke are less likely to respond to treatment with methotrexate and tumor necrosis factor inhibitors: observations from the Epidemiological Investigation of Rheumatoid Arthritis and the Swedish Rheumatology Register cohorts. Arthritis Rheum.

[13] Söderlin MK, Petersson IF, Geborek P (2012). The effect of smoking on response and drug survival in rheumatoid arthritis patients treated with their first anti-TNF drug. Scand J Rheumatol..

[14] Canhão H, Rodrigues AM, Mourão AF, Martins F, Santos MJ, Canas-Silva J (2012). Comparative effectiveness and predictors of response to tumour necrosis factor inhibitor therapies in rheumatoid arthritis. Rheumatology (Oxford).

[15] Hyrich KL, Watson KD, Silman AJ, Symmons DPM, British Society for Rheumatology Biologics Register (2006). Predictors of response to anti-TNF-alpha therapy among patients with rheumatoid arthritis: results from the British Society for Rheumatology Biologics Register. Rheumatology (Oxford).

[16] McWilliams DF, Walsh DA (2016). Factors predicting pain and early discontinuation of tumour necrosis factor-α-inhibitors in people with rheumatoid arthritis: results from the British Society for Rheumatology Biologics Register. BMC Musculoskelet Disord..

[17] Mancarella L, Bobbio-Pallavicini F, Ceccarelli F, Falappone PC, Ferrante A, Malesci D (2007). Good clinical response, remission, and predictors of remission in rheumatoid arthritis patients treated with tumor necrosis factor-alpha blockers: the GISEA study. J Rheumatol..

[18] Daïen CI, Morel J. Predictive factors of response to biological disease modifying antirheumatic drugs: towards personalized medicine. Mediators Inflamm. 2014;2014:386148, 11 pages. 10.1155/2014/386148 PMC3913459 24523570

[19] Nair SC, Welsing PMJ, Choi IYK, Roth J, Holzinger D, Bijlsma JWJ (2016). A personalized approach to biological therapy using prediction of clinical response based on MRP8/14 serum complex levels in rheumatoid arthritis patients. PLoS One..

[20] Isaacs JD, Cohen SB, Emery P, Tak PP, Wang J, Lei G (2012). Effect of baseline rheumatoid factor and anticitrullinated peptide antibody serotype on rituximab clinical response: a meta-analysis. Ann Rheum Dis..

[21] Buch MH, Smolen JS, Betteridge N, Breedveld FC, Burmester G, Dörner T (2011). Updated consensus statement on the use of rituximab in patients with rheumatoid arthritis. Ann Rheum Dis..

[22] Chatzidionysiou K, Lie E, Nasonov E, Lukina G, Hetland ML, Tarp U (2011). Highest clinical effectiveness of rituximab in autoantibody-positive patients with rheumatoid arthritis and in those for whom no more than one previous TNF antagonist has failed: pooled data from 10 European registries. Ann Rheum Dis..

[23] Maneiro RJ, Salgado E, Carmona L, Gomez-Reino JJ (2013). Rheumatoid factor as predictor of response to abatacept, rituximab and tocilizumab in rheumatoid arthritis: systematic review and meta-analysis. Semin Arthritis Rheum..

[24] Potter C, Hyrich KL, Tracey A, Lunt M, Plant D, Symmons DPM (2009). Association of rheumatoid factor and anti-cyclic citrullinated peptide positivity, but not carriage of shared epitope or PTPN22 susceptibility variants, with anti-tumour necrosis factor response in rheumatoid arthritis. Ann Rheum Dis..

[25] Lv Q, Yin Y, Li X, Shan G, Wu X, Liang D, et al. The status of rheumatoid factor and anti-cyclic citrullinated peptide antibody are not associated with the effect of anti-TNF a agent treatment in patients with rheumatoid arthritis: a meta-analysis. PLoS One. 2014;9:e89442. 10.1371/journal.pone.0089442 PMC3937352 24586782

[26] Gottenberg JE, Courvoisier DS, Hernandez MV, Iannone F, Lie E, Canhão H (2016). Brief Report: Association of rheumatoid factor and anti-citrullinated protein antibody positivity with better effectiveness of abatacept: results from the Pan-European Registry Analysis. Arthritis Rheumatol..

[27] Kawashiri S-Y, Kawakami A, Iwamoto N, Fujikawa K, Aramaki T, Tamai M (2011). In rheumatoid arthritis patients treated with tocilizumab, the rate of clinical disease activity index (CDAI) remission at 24 weeks is superior in those with higher titers of IgM-rheumatoid factor at baseline. Mod Rheumatol..

[28] Pers Y-M, Fortunet C, Constant E, Lambert J, Godfrin-Valnet M, De Jong A (2014). Predictors of response and remission in a large cohort of rheumatoid arthritis patients treated with tocilizumab in clinical practice. Rheumatology (Oxford).

[29] Kubo S, Nakayamada S, Nakano K, Hirata S, Fukuyo S, Miyagawa I (2016). Comparison of the efficacies of abatacept and tocilizumab in patients with rheumatoid arthritis by propensity score matching. Ann Rheum Dis..

[30] Ishiguro N, Atsumi T, Harigai M, Mimori T, Nishimoto N, Sumida T, et al. Effectiveness and safety of tocilizumab in achieving clinical and functional remission, and sustaining efficacy in biologics-naive patients with rheumatoid arthritis: The FIRST Bio study. Mod Rheumatol. 2017;27:217–26. 10.1080/14397595.2016.1206507 27414105

[31] Inciarte-Mundo J, Ramirez J, Hernández MV, Ruiz-Esquide V, Cuervo A, Cabrera-Villalba SR, et al. Calprotectin and TNF trough serum levels identify power Doppler ultrasound synovitis in rheumatoid arthritis and psoriatic arthritis patients in remission or with low disease activity. Arthritis Res Ther. 2016;18:160. 10.1186/s13075-016-1032-z PMC4938924 27391315

[32] Abildtrup M, Kingsley GH, Scott DL (2015). Calprotectin as a biomarker for rheumatoid arthritis: a systematic review. J Rheumatol..

[33] Inciarte-Mundo J, Ruiz-Esquide V, Hernandez MV, Canete JD, Cabrera-Villalba SR, Ramirez J (2015). Calprotectin more accurately discriminates the disease status of rheumatoid arthritis patients receiving tocilizumab than acute phase reactants. Rheumatol (United Kingdom).

[34] Choi IY, Gerlag DM, Herenius MJ, Thurlings RM, Wijbrandts C a, Foell D (2015). MRP8/14 serum levels as a strong predictor of response to biological treatments in patients with rheumatoid arthritis. Ann Rheum Dis.

[35] Raterman HG, Vosslamber S, de Ridder S, Nurmohamed MT, Lems WF, Boers M (2012). The interferon type I signature towards prediction of non-response to rituximab in rheumatoid arthritis patients. Arthritis Res Ther..

[36] Thurlings RM, Boumans M, Tekstra J, Van Roon JA, Vos K, Van Westing DM (2010). Relationship between the type I interferon signature and the response to rituximab in rheumatoid arthritis patients. Arthritis Rheum..

[37] Hogan VE, Holweg CTJ, Choy DF, Kummerfeld SK, Hackney JA, Teng YKO (2012). Pretreatment synovial transcriptional profile is associated with early and late clinical response in rheumatoid arthritis patients treated with rituximab. Ann Rheum Dis..

[38] Wright HL, Thomas HB, Moots RJ, Edwards SW (2014). Interferon gene expression signature in rheumatoid arthritis neutrophils correlates with a good response to TNFi therapy. Rheumatology (Oxford).

[39] Mavragani CP, La DT, Stohl W, Crow MK (2010). Association of the response to tumor necrosis factor antagonists with plasma type I interferon activity and interferon-beta/alfa ratios in rheumatoid arthritis patients: a post hoc analysis of a predominantly hispanic cohort. Arthritis Rheum..

[40] Sekiguchi N, Kawauchi S, Furuya T, Inaba N, Matsuda K, Ando S, et al. Messenger ribonucleic acid expression profile in peripheral blood cells from RA patients following treatment with an anti-TNF-alfa monoclonal antibody, infliximab. Rheumatology (Oxford). 2008;47:780–8. 10.1093/rheumatology/ken083 18388148

[41] van Baarsen LG, Wijbrandts CA, Rustenburg F, Cantaert T, van der Pouw Kraan TC, Baeten DL (2010). Regulation of IFN response gene activity during infliximab treatment in rheumatoid arthritis is associated with clinical response to treatment. Arthritis Res Ther..

[42] Sanayama Y, Ikeda K, Saito Y, Kagami S-I, Yamagata M, Furuta S (2014). Prediction of therapeutic responses to tocilizumab in patients with rheumatoid arthritis: biomarkers identified by analysis of gene expression in peripheral blood mononuclear cells using genome-wide DNA microarray. Arthritis Rheumatol (Hoboken, NJ).

[43] Crow MK (2010). Type I interferon in organ-targeted autoimmune and inflammatory diseases. Arthritis Res Ther.

[44] Adriaansen J, Kuhlman RR, Van Holten J, Kaynor C, Vervoordeldonk MJBM, Tak DPP (2006). Intraarticular interferon-β gene therapy ameliorates adjuvant arthritis in rats. Hum Gene Ther..

[45] van Holten J, Reedquist K, Sattonet-Roche P, Smeets TJM, Plater-Zyberk C, Vervoordeldonk MJ (2004). Treatment with recombinant interferon-beta reduces inflammation and slows cartilage destruction in the collagen-induced arthritis model of rheumatoid arthritis. Arthritis Res Ther..

[46] Treschow AP, Teige I, Nandakumar KS, Holmdahl R, Issazadeh-Navikas S (2005). Stromal cells and osteoclasts are responsible for exacerbated collagen-induced arthritis in interferon-beta-deficient mice. Arthritis Rheum..

[47] van Holten J (2005). Expression of interferon in synovial tissue from patients with rheumatoid arthritis: comparison with patients with osteoarthritis and reactive arthritis. Ann Rheum Dis..

[48] Smeets TJM, Dayer JM, Kraan MC, Versendaal J, Chicheportiche R, Breedveld FC (2000). The effects of interferon-beta treatment on synovial inflammation and expression of metalloproteinases in patients with rheumatoid arthritis. Arthritis Rheum..

[49] Tak PP (2004). IFN-beta in rheumatoid arthritis. Front Biosci..

[50] Oliver J, Plant D, Webster AP, Barton A (2015). Genetic and genomic markers of anti-TNF treatment response in rheumatoid arthritis. Biomark Med..

[51] Montes Marquez A, Ferreiro-Iglesias A, Davila-Fajardo C, Pascual-Salcedo D, Perez-Pampin E, Moreno-Ramos M (2014). Lack of validation of genetic variants associated with anti-tumor necrosis factor therapy response in rheumatoid arthritis: a genome-wide association study replication and meta-analysis. Arthritis Res Ther..

[52] Plant D, Bowes J, Potter C, Hyrich KL, Morgan AW, Wilson AG (2011). Genome-wide association study of genetic predictors of anti-tumor necrosis factor treatment efficacy in rheumatoid arthritis identifies associations with polymorphisms at seven loci. Arthritis Rheum..

[53] Krintel SB, Palermo G, Johansen JS, Germer S, Essioux L, Benayed R (2012). Investigation of single nucleotide polymorphisms and biological pathways associated with response to TNFα inhibitors in patients with rheumatoid arthritis. Pharmacogenet Genomics..

[54] Smith SL, Plant D, Lee XH, Massey J, Hyrich K, Morgan AW (2016). Previously reported *PDE3A–SLCO1C1* genetic variant does not correlate with anti-TNF response in a large UK rheumatoid arthritis cohort. Pharmacogenomics..

[55] Acosta-Colman I, Palau N, Tornero J, Fernández-Nebro A, Blanco F, González-Alvaro I (2013). GWAS replication study confirms the association of PDE3A-SLCO1C1 with anti-TNF therapy response in rheumatoid arthritis. Pharmacogenomics..

[56] Cui J, Stahl EA, Saevarsdottir S, Miceli C, Diogo D, Trynka G, et al. Genome-wide association study and gene expression analysis identifies CD84 as a predictor of response to etanercept therapy in rheumatoid arthritis. PLoS Genet. 2013;9:e1003394. 10.1371/journal.pgen.1003394 PMC3610685 23555300

[57] Cui J, Saevarsdottir S, Thomson B, Padyukov L, Van Der Helm-van Mil AHM, Nititham J (2010). Rheumatoid arthritis risk allele PTPRC is also associated with response to anti-tumor necrosis factor α therapy. Arthritis Rheum..

[58] Plant D, Prajapati R, Hyrich KL, Morgan AW, Wilson AG, Isaacs JD (2012). Replication of association of the PTPRC gene with response to anti-tumor necrosis factor therapy in a large UK cohort. Arthritis Rheum..

[59] Ferreiro-Iglesias A, Montes A, Perez-Pampin E, Cañete JD, Raya E, Magro-Checa C (2016). Replication of PTPRC as genetic biomarker of response to TNF inhibitors in patients with rheumatoid arthritis. Pharmacogenomics J..

[60] Pappas DA, Oh C, Plenge RM, Kremer JM, Greenberg JD (2013). Association of rheumatoid arthritis risk alleles with response to anti-TNF biologics: results from the CORRONA registry and meta-analysis. Inflammation..

[61] Sieberts SK, Zhu F, García-García J, Stahl E, Pratap A, Pandey G (2016). Crowdsourced assessment of common genetic contribution to predicting anti-TNF treatment response in rheumatoid arthritis. Nat Commun..

[62] Smith SL, Plant D, Eyre S, Barton A (2013). The potential use of expression profiling: implications for predicting treatment response in rheumatoid arthritis. Ann Rheum Dis..

[63] Burska a N, Roget K, Blits M, Soto Gomez L, van de Loo F, Hazelwood LD (2014). Gene expression analysis in RA: towards personalized medicine. Pharmacogenomics J.

[64] Kelly S, Humby F, Filer A, Ng N, Di Cicco M, Hands RE (2013). Ultrasound-guided synovial biopsy: a safe, well-tolerated and reliable technique for obtaining high-quality synovial tissue from both large and small joints in early arthritis patients. Ann Rheum Dis..

[65] Klein K, Ospelt C, Gay S (2012). Epigenetic contributions in the development of rheumatoid arthritis. Arthritis Res Ther..

[66] Ammari M, Jorgensen C, Apparailly F (2013). Impact of microRNAs on the understanding and treatment of rheumatoid arthritis. Curr Opin Rheumatol..

[67] Castro-Villegas C, Pérez-Sánchez C, Escudero A, Filipescu I, Verdu M, Ruiz-Limón P (2011). Circulating miRNAs as potential biomarkers of therapy effectiveness in rheumatoid arthritis patients treated with anti-TNFα. Arthritis Res Ther..

[68] Cuppen B v, Rossato M, Fritsch-Stork R, Concepcion A, Schenk Y, Bijlsma J, et al. Can baseline serum microRNAs predict response to TNF-alpha inhibitors in rheumatoid arthritis? Arthritis Res Ther. 2016;18:189. 10.1186/s13075-016-1085-z PMC4997731 27558398

[69] Krintel SB, Dehlendorff C, Hetland ML, Hørslev-Petersen K, Andersen KK, Junker P, et al. Prediction of treatment response to adalimumab: a double-blind placebo-controlled study of circulating microRNA in patients with early rheumatoid arthritis. Pharmacogenomics J. 2016;16:141–6. 10.1038/tpj.2015.30 25939484

[70] Dennis G, Holweg CT, Kummerfeld SK, Choy DF, Setiadi A, Hackney JA (2014). Synovial phenotypes in rheumatoid arthritis correlate with response to biologic therapeutics. Arthritis Res Ther..

[71] Bresnihan B, Gerlag DM, Rooney T, Smeets TJM, Wijbrandts CA, Boyle D (2007). Synovial macrophages as a biomarker of response to therapeutic intervention in rheumatoid arthritis: standardization and consistency across centers. J Rheumatol..

[72] van der Pouw Kraan TC, Wijbrandts C a, van Baarsen LG, Rustenburg F, Baggen JM, Verweij CL (2008). Responsiveness to anti-tumour necrosis factor alpha therapy is related to pre-treatment tissue inflammation levels in rheumatoid arthritis patients. Ann Rheum Dis.

[73] Wijbrandts C a, Dijkgraaf MGW, Kraan MC, Vinkenoog M, Smeets TJ, Dinant H (2008). The clinical response to infliximab in rheumatoid arthritis is in part dependent on pretreatment tumour necrosis factor alpha expression in the synovium. Ann Rheum Dis.

[74] Badot V, Galant C, Nzeusseu Toukap A, Theate I, Maudoux A-L, Van den Eynde BJ (2009). Gene expression profiling in the synovium identifies a predictive signature of absence of response to adalimumab therapy in rheumatoid arthritis. Arthritis Res Ther..

[75] Cañete JD, Celis R, Moll C, Izquierdo E, Marsal S, Sanmartí R (2009). Clinical significance of synovial lymphoid neogenesis and its reversal after anti-tumour necrosis factor alpha therapy in rheumatoid arthritis. Ann Rheum Dis..

[76] Wright HL, Bucknall RC, Moots RJ, Edwards SW. Analysis of SF and plasma cytokines provides insights into the mechanisms of inflammatory arthritis and may predict response to therapy. Rheumatology (Oxford). 2012;51:451–9. 10.1093/rheumatology/ker338 22179732

[77] Klaasen R, Thurlings RM, Wijbrandts CA, Van Kuijk AW, Baeten D, Gerlag DM (2009). The relationship between synovial lymphocyte aggregates and the clinical response to infliximab in rheumatoid arthritis: a prospective study. Arthritis Rheum..

[78] Van Baarsen LGM, Wijbrandts CA, Timmer TCG, Van Der Pouw KTCTM, Tak PP, Verweij CL (2010). Synovial tissue heterogeneity in rheumatoid arthritis in relation to disease activity and biomarkers in peripheral blood. Arthritis Rheum..

[79] Pachot A, Arnaud B, Marrote H, Cazalis M-A, Diasparra J, Gouraud A (2007). Increased tumor necrosis factor-alpha mRNA expression in whole blood from patients with rheumatoid arthritis: reduction after infliximab treatment does not predict response. J Rheumatol..

[80] Fabre S, Dupuy AM, Dossat N, Guisset C, Cohen JD, Cristol JP (2008). Protein biochip array technology for cytokine profiling predicts etanercept responsiveness in rheumatoid arthritis. Clin Exp Immunol..

[81] Chen D-Y, Chen Y-M, Chen H-H, Hsieh C-W, Lin C-C, Lan J-L (2011). Increasing levels of circulating Th17 cells and interleukin-17 in rheumatoid arthritis patients with an inadequate response to anti-TNF-α therapy. Arthritis Res Ther..

[82] Marotte H, Maslinski W, Miossec P (2005). Circulating tumour necrosis factor-alpha bioactivity in rheumatoid arthritis patients treated with infliximab: link to clinical response. Arthritis Res Ther..

[83] Kayakabe K, Kuroiwa T, Sakurai N, Ikeuchi H, Kadiombo AT, Sakairi T (2012). Interleukin-1β measurement in stimulated whole blood cultures is useful to predict response to anti-TNF therapies in rheumatoid arthritis. Rheumatology (Oxford).

[84] Meusch U, Krasselt M, Rossol M, Baerwald C, Klingner M, Wagner U (2015). In vitro response pattern of monocytes after tmTNF reverse signaling predicts response to anti-TNF therapy in rheumatoid arthritis. J Transl Med..

[85] Alzabin S, Abraham SM, Taher TE, Palfreeman a, Hull D, McNamee K (2012). Incomplete response of inflammatory arthritis to TNF blockade is associated with the Th17 pathway. Ann Rheum Dis.

[86] Visvanathan S, Marini JC, Smolen JS, St. Clair EW, Pritchard C, Shergy W (2007). Changes in biomarkers of inflammation and bone turnover and associations with clinical efficacy following infliximab plus methotrexate therapy in patients with early rheumatoid arthritis. J Rheumatol..

[87] Lequerré T, Jouen F, Brazier M, Clayssens S, Klemmer N, Ménard JF, et al. Autoantibodies, metalloproteinases and bone markers in rheumatoid arthritis patients are unable to predict their responses to infliximab. Rheumatology (Oxford). 2007;46:446–53. 10.1093/rheumatology/kel262 16899502

[88] Visvanathan S, Rahman MU, Keystone E, Genovese M, Klareskog L, Hsia E (2010). Association of serum markers with improvement in clinical response measures after treatment with golimumab in patients with active rheumatoid arthritis despite receiving methotrexate: results from the GO-FORWARD study. Arthritis Res Ther..

[89] Morozzi G, Fabbroni M, Bellisai F, Cucini S, Simpatico A, Galeazzi M (2007). Low serum level of COMP, a cartilage turnover marker, predicts rapid and high ACR70 response to adalimumab therapy in rheumatoid arthritis. Clin Rheumatol..

[90] González-Alvaro I, Ortiz AM, Tomero EG, Balsa A, Orte J, Laffon A (2007). Baseline serum RANKL levels may serve to predict remission in rheumatoid arthritis patients treated with TNF antagonists. Ann Rheum Dis..

[91] Cuppen BVJ, Fu J, van Wietmarschen HA, Harms AC, Koval S, Marijnissen ACA (2016). Exploring the inflammatory metabolomic profile to predict response to TNF-α inhibitors in rheumatoid arthritis. PLoS One..

[92] Priori R, Casadei L, Valerio M, Scrivo R, Valesini G, Manetti C (2015). 1H-NMR-based metabolomic study for identifying serum profiles associated with the response to etanercept in patients with rheumatoid arthritis. PLoS One..

[93] Kapoor SR, Filer A, Fitzpatrick MA, Fisher BA, Taylor PC, Buckley CD (2013). Metabolic profiling predicts response to anti-tumor necrosis factor-alfa therapy in patients with rheumatoid arthritis. Arthritis Rheum..

[94] Sellam J, Hendel-Chavez H, Rouanet S, Abbed K, Combe B, Le Loët X (2011). B cell activation biomarkers as predictive factors for the response to rituximab in rheumatoid arthritis a six-month, national, multicenter, open-label study. Arthritis Rheum..

[95] Ferraccioli G, Tolusso B, Bobbio-Pallavicini F, Gremese E, Ravagnani V, Benucci M (2012). Biomarkers of good EULAR response to the B cell depletion therapy in all seropositive rheumatoid arthritis patients: Clues for the pathogenesis. PLoS One..

[96] Sellam J, Rouanet S, Hendel-Chavez H, Miceli-Richard C, Combe B, Sibilia J (2013). CCL19, a B cell chemokine, is related to the decrease of blood memory B cells and predicts the clinical response to rituximab in patients with rheumatoid arthritis. Arthritis Rheum..

[97] Teng YKO, Levarht EWN, Hashemi M, Bajema IM, Toes REM, Huizinga TWJ (2007). Immunohistochemical analysis as a means to predict responsiveness to rituximab treatment. Arthritis Rheum..

[98] Stradner MH, Dejaco C, Brickmann K, Graninger WB, Brezinschek HP (2016). A combination of cellular biomarkers predicts failure to respond to rituximab in rheumatoid arthritis: a 24-week observational study. Arthritis Res Ther..

[99] Vital EM, Dass S, Rawstron AC, Buch MH, Goëb V, Henshaw K (2010). Management of nonresponse to rituximab in rheumatoid arthritis: predictors and outcome of re-treatment. Arthritis Rheum..

[100] Brezinschek H-P, Rainer F, Brickmann K, Graninger WB (2012). B lymphocyte-typing for prediction of clinical response to rituximab. Arthritis Res Ther..

[101] Owczarczyk K, Lal P, Abbas AR, Wolslegel K, Holweg CTJ, Dummer W (2011). A plasmablast biomarker for nonresponse to antibody therapy to CD20 in rheumatoid arthritis. Sci Transl Med.

[102] Dass S, Rawstron AC, Vital EM, Henshaw K, McGonagle D, Emery P (2008). Highly sensitive B cell analysis predicts response to rituximab therapy in rheumatoid arthritis. Arthritis Rheum..

[103] Vital EM, Dass S, Buch MH, Rawstron AC, Emery P. An extra dose of rituximab improves clinical response in rheumatoid arthritis patients with initial incomplete B cell depletion: a randomised controlled trial. Ann Rheum Dis. 2015;74:1195–201. 10.1136/annrheumdis-2013-204544 24443001

[104] Breedveld F, Agarwal S, Yin M, Ren S, Li NF, Shaw TM (2007). Rituximab pharmacokinetics in patients with rheumatoid arthritis: B-cell levels do not correlate with clinical response. J Clin Pharmacol..

[105] Kavanaugh A, Rosengren S, Lee SJ, Hammaker D, Firestein GS, Kalunian K (2008). Assessment of rituximab’s immunomodulatory synovial effects (ARISE trial). 1: clinical and synovial biomarker results. Ann Rheum Dis.

[106] Roll P, Dörner T, Tony H-P (2008). Anti-CD20 therapy in patients with rheumatoid arthritis: predictors of response and B cell subset regeneration after repeated treatment. Arthritis Rheum..

[107] Leandro MJ, Cambridge G, Ehrenstein MR, Edwards JCW (2006). Reconstitution of peripheral blood B cells after depletion with rituximab in patients with rheumatoid arthritis. Arthritis Rheum..

[108] Sellam J, Rouanet S, Hendel-Chavez H, Abbed K, Sibilia J, Tebib J (2011). Blood memory B cells are disturbed and predict the response to rituximab in patients with rheumatoid arthritis. Arthritis Rheum..

[109] Nakou M, Katsikas G, Sidiropoulos P, Bertsias G, Papadimitraki E, Raptopoulou A (2009). Rituximab therapy reduces activated B cells in both the peripheral blood and bone marrow of patients with rheumatoid arthritis: depletion of memory B cells correlates with clinical response. Arthritis Res Ther..

[110] Möller B, Aeberli D, Eggli S, Fuhrer M, Vajtai I, Vögelin E (2009). Class-switched B cells display response to therapeutic B-cell depletion in rheumatoid arthritis. Arthritis Res Ther..

[111] Adlowitz DG, Barnard J, Biear JN, Cistrone C, Owen T, Wang W, et al. Expansion of activated peripheral blood memory B cells in rheumatoid arthritis, impact of B cell depletion therapy, and biomarkers of response. PLoS One. 2015;10:e0128269. 10.1371/journal.pone.0128269 PMC4457888 26047509

[112] Trouvin AP, Jacquot S, Grigioni S, Curis E, Dedreux I, Roucheux A (2015). Usefulness of monitoring of B cell depletion in rituximab-treated rheumatoid arthritis patients in order to predict clinical relapse: a prospective observational study. Clin Exp Immunol..

[113] Thurlings RM, Vos K, Wijbrandts C a, Zwinderman a H, Gerlag DM, Tak PP (2008). Synovial tissue response to rituximab: mechanism of action and identification of biomarkers of response. Ann Rheum Dis.

[114] Vos K, Thurlings RM, Wijbrandts CA, Van Schaardenburg D, Gerlag DM, Tak PP (2007). Early effects of rituximab on the synovial cell infiltrate in patients with rheumatoid arthritis. Arthritis Rheum..

[115] Rosengren S, Wei N, Kalunian KC, Zvaifler NJ, Kavanaugh A, Boyle DL (2008). Elevated autoantibody content in rheumatoid arthritis synovia with lymphoid aggregates and the effect of rituximab. Arthritis Res Ther..

[116] Care MA, Stephenson SJ, Barnes NA, Fan I, Zougman A, El-sherbiny YM, et al. Network analysis identifies proinflammatory plasma cell polarization for secretion of ISG15 in human autoimmunity. J Immunol. 2016;197:1447–59. 10.4049/jimmunol.1600624 PMC4974491 27357150

[117] R4-RA Clinical Trial. http://www.r4ra-nihr.whri.qmul.ac.uk/.

[118] Vosslamber S, Raterman HG, van der Pouw KTCTM, Schreurs MWJ, von Blomberg BME, Nurmohamed MT (2011). Pharmacological induction of interferon type I activity following treatment with rituximab determines clinical response in rheumatoid arthritis. Ann Rheum Dis..

[119] Sellam J, Marion-Thore S, Dumont F, Jacques S, Garchon HJ, Rouanet S (2014). Use of whole-blood transcriptomic profiling to highlight several pathophysiologic pathways associated with response to rituximab in patients with rheumatoid arthritis: data from a randomized, controlled, open-label trial. Arthritis Rheumatol..

[120] Gutierrez-Roelens I, Galant C, Theate I, Lories RJ, Durez P, Nzeusseu-Toukap A (2011). Rituximab treatment induces the expression of genes involved in healing processes in the rheumatoid arthritis synovium. Arthritis Rheum..

[121] Lee YH, Bae SC, Song GG (2014). Functional FCGR3A 158 V/F and IL-6 − 174 C/G polymorphisms predict response to biologic therapy in patients with rheumatoid arthritis: a meta-analysis. Rheumatol Int..

[122] Ruyssen-Witrand A, Rouanet S, Combe B, Dougados M, Le Loët X, Sibilia J (2013). Association between –871C > T promoter polymorphism in the B-cell activating factor gene and the response to rituximab in rheumatoid arthritis patients. Rheumatology (Oxford).

[123] Duroux-Richard I, Pers YM, Fabre S, Ammari M, Baeten D, Cartron G, et al. Circulating miRNA-125b is a potential biomarker predicting response to rituximab in rheumatoid arthritis. Mediators Inflamm. 2014;2014:342524, 9 pages. 10.1155/2014/342524 PMC3980876 24778468

[124] Fabre S, Guisset C, Tatem L, Dossat N, Dupuy AM, Cohen JD (2009). Protein biochip array technology to monitor rituximab in rheumatoid arthritis. Clin Exp Immunol..

[125] Buch MH, Vital EM, Emery P (2008). Abatacept in the treatment of rheumatoid arthritis. Arthritis Res Ther.

[126] Buch MH, Boyle DL, Rosengren S, Saleem B, Reece RJ, Rhodes L (2009). Mode of action of abatacept in rheumatoid arthritis patients having failed tumour necrosis factor blockade: a histological, gene expression and dynamic magnetic resonance imaging pilot study. Ann Rheum Dis.

[127] Scarsi M, Paolini L, Ricotta D, Pedrini A, Piantoni S, Caimi L (2014). Abatacept reduces levels of switched memory B cells, autoantibodies, and immunoglobulins in patients with rheumatoid arthritis. J Rheumatol..

[128] Pieper J, Herrath J, Raghavan S, Muhammad K, Vollenhoven R, Malmstrom V (2013). CTLA4-Ig (abatacept) therapy modulates T cell effector functions in autoantibody-positive rheumatoid arthritis patients. BMC Immunol..

[129] Iwata S, Nakayamada S, Fukuyo S, Kubo S, Yunoue N, Wang SP (2015). Activation of syk in peripheral blood B cells in patients with rheumatoid arthritis: a potential target for abatacept therapy. Arthritis Rheumatol..

[130] Picchianti Diamanti A, Rosado MM, Scarsella M, Germano V, Giorda E, Cascioli S (2014). Abatacept (cytotoxic T lymphocyte antigen 4-immunoglobulin) improves B cell function and regulatory T cell inhibitory capacity in rheumatoid arthritis patients non-responding to anti-tumour necrosis factor-alfa agents. Clin Exp Immunol..

[131] Scarsi M, Ziglioli T, Airo P (2011). Baseline numbers of circulating CD28-negative T cells may predict clinical response to abatacept in patients with rheumatoid arthritis. J Rheumatol..

[132] Weyand CM, Fulbright JW, Goronzy JJ (2003). Immunosenescence, autoimmunity, and rheumatoid arthritis. Exp Gerontol..

[133] Nakamura S, Suzuki K, Iijima H, Hata Y, Lim CR, Ishizawa Y (2016). Identification of baseline gene expression signatures predicting therapeutic responses to three biologic agents in rheumatoid arthritis: a retrospective observational study. Arthritis Res Ther..

[134] Umemura M, Isozaki T, Ishii S, Seki S, Oguro N, Miura Y (2014). Reduction of serum ADAM17 level accompanied with decreased cytokines after abatacept therapy in patients with rheumatoid arthritis. Int J Biomed Sci..

[135] Enevold C, Baslund B, Linde L, Josephsen NL, Tarp U, Lindegaard H (2014). Interleukin-6-receptor polymorphisms rs12083537, rs2228145, and rs4329505 as predictors of response to tocilizumab in rheumatoid arthritis. Pharmacogenet Genomics..

[136] Wang J, Platt A, Upmanyu R, Germer S, Lei G, Rabe C (2013). IL-6 pathway-driven investigation of response to IL-6 receptor inhibition in rheumatoid arthritis. BMJ Open..

[137] Wang J, Bansal a T, Martin M, Germer S, Benayed R, Essioux L (2013). Genome-wide association analysis implicates the involvement of eight loci with response to tocilizumab for the treatment of rheumatoid arthritis. Pharmacogenomics J.

[138] Maldonado-Montoro M, Cañadas-Garre M, González-Utrilla A, Plaza-Plaza JC, Calleja-Hernández MÁ. Genetic and clinical biomarkers of tocilizumab response in patients with rheumatoid arthritis. Pharmacol Res. 2016;111:264–27. 10.1016/j.phrs.2016.06.016 27339827

[139] Mesko B, Poliska S, Szamosi S, Szekanecz Z, Podani J, Varadi C (2012). Peripheral blood gene expression and IgG glycosylation profiles as markers of tocilizumab treatment in rheumatoid arthritis. J Rheumatol..

[140] Ducreux J, Durez P, Galant C, Toukap AN, Van Den Eynde B, Houssiau FA (2014). Global molecular effects of tocilizumab therapy in rheumatoid arthritis synovium. Arthritis Rheumatol..

[141] De Groof A, Ducreux J, Humby F, Nzeusseu Toukap A, Badot V, Pitzalis C (2016). Higher expression of TNFα-induced genes in the synovium of patients with early rheumatoid arthritis correlates with disease activity, and predicts absence of response to first line therapy. Arthritis Res Ther..

[142] Shimamoto K, Ito T, Ozaki Y, Amuro H, Tanaka A, Nishizawa T (2013). Serum interleukin 6 before and after therapy with tocilizumab is a principal biomarker in patients with rheumatoid arthritis. J Rheumatol..

[143] Uno K, Yoshizaki K, Iwahashi M, Yamana J, Yamana S, Tanigawa M (2015). Pretreatment prediction of individual rheumatoid arthritis patients’ response to anti-cytokine therapy using serum cytokine/chemokine/soluble receptor biomarkers. PLoS One..

[144] Das S, Vital EM, Horton S, Bryer D, El-Sherbiny Y, Rawstron AC (2014). Abatacept or tocilizumab after rituximab in rheumatoid arthritis? An exploratory study suggests non-response to rituximab is associated with persistently high IL-6 and better clinical response to IL-6 blocking therapy. Ann Rheum Dis..

[145] Lee SJ, Park W, Park SH, Shim SC, Baek HJ, Yoo DH, et al. Low baseline interleukin-17A levels are associated with better treatment response at 12 weeks to tocilizumab therapy in rheumatoid arthritis patients. J Immunol Res. 2015;2015:487230, 7 pages. 10.1155/2015/487230 PMC4398953 25922848

[146] Mahmood Z, Muhammad K, Schmalzing M, Roll P, Dörner T, Tony H-P (2015). CD27–IgD– memory B cells are modulated by in vivo interleukin-6 receptor (IL-6R) blockade in rheumatoid arthritis. Arthritis Res Ther..

[147] Kikuchi J, Hashizume M, Kaneko Y, Yoshimoto K, Nishina N, Takeuchi T (2015). Peripheral blood CD4 + CD25 + CD127low regulatory T cells are significantly increased by tocilizumab treatment in patients with rheumatoid arthritis: increase in regulatory T cells correlates with clinical response. Arthritis Res Ther..

[148] Early Breast Cancer Trialists’ Collaborative Group (EBCTCG), Dowsett M, Forbes JF, Bradley R, Ingle J, Aihara T, et al. Aromatase inhibitors versus tamoxifen in early breast cancer: patient-level meta-analysis of the randomised trials. Lancet. 2015;386:1341–52. 10.1016/S0140-6736(15)61074-1 26211827

[149] Whitaker JW, Boyle DL, Bartok B, Ball ST, Gay S, Wang W (2015). Integrative omics analysis of rheumatoid arthritis identifies non-obvious therapeutic targets. PLoS One..

[150] Hueber W, Tomooka BH, Batliwalla F, Li W, Monach P a, Tibshirani RJ (2009). Blood autoantibody and cytokine profiles predict response to anti-tumor necrosis factor therapy in rheumatoid arthritis. Arthritis Res Ther.

[151] Obry A, Hardouin J, Lequerré T, Jarnier F, Boyer O, Fardellone P (2015). Identification of 7 proteins in sera of RA patients with potential to predict ETA/MTX treatment response. Theranostics..

[152] Ortea I, Roschitzki B, Ovalles JG, Longo JL, de la Torre I, González I (2012). Discovery of serum proteomic biomarkers for prediction of response to infliximab (a monoclonal anti-TNF antibody) treatment in rheumatoid arthritis: an exploratory analysis. J Proteomics..

[153] Trocmé C, Marotte H, Baillet a, Pallot-Prades B, Garin J, Grange L (2009). Apolipoprotein A-I and platelet factor 4 are biomarkers for infliximab response in rheumatoid arthritis. Ann Rheum Dis.

[154] Hambardzumyan K, Bolce RJ, Saevarsdottir S, Forslind K, Wallman JK, Cruickshank SE (2016). Association of a multibiomarker disease activity score at multiple time-points with radiographic progression in rheumatoid arthritis: results from the SWEFOT trial. RMD Open..

[155] Ortea I, Roschitzki B, López-Rodríguez R, Tomero EG, Ovalles JG, López-Longo J (2016). Independent candidate serum protein biomarkers of response to adalimumab and to infliximab in rheumatoid arthritis: an exploratory study. PLoS One..

